# Clinical burden of hyperphagia, obesity and health outcomes in paediatric individuals with Bardet‐Biedl syndrome: A CRIBBS data analysis

**DOI:** 10.1111/ijpo.70026

**Published:** 2025-06-02

**Authors:** Jeremy Pomeroy, Uzoma Okorie, Jesse G. Richardson, Ariane Faucher, Raluca Ionescu‐Ittu, Francis Vekeman, Caroline Huber, Robert M. Haws

**Affiliations:** ^1^ Fritz Wenzel Center for Clinical Research, Marshfield Clinic Research Institute Marshfield Wisconsin USA; ^2^ Department of Pediatrics Marshfield Clinic Research Institute Marshfield Wisconsin USA; ^3^ Office of Research Computing & Analytics, Marshfield Clinic Research Institute Marshfield Wisconsin USA; ^4^ STATLOG Inc. Montreal Quebec Canada; ^5^ Rhythm Pharmaceuticals, Inc. Boston Massachusetts USA

**Keywords:** Bardet‐Biedl syndrome, comorbidities, disease burden, hunger, natural history, obesity

## Abstract

**Background:**

To date, research on the natural history of hyperphagia, weight gain and health outcomes in individuals with Bardet‐Biedl syndrome (BBS) has been limited. Understanding these characteristics is important for disease burden management.

**Objectives:**

The international Clinical Registry Investigating BBS (CRIBBS) is a registry of individuals with BBS to investigate the severity of hyperphagia, the natural history of weight gain and the frequency of obesity‐associated health outcomes in paediatric individuals with BBS.

**Methods:**

Paediatric participants (<18 years of age) enrolled in CRIBBS were evaluated to assess weight and weight loss interventions, hyperphagia and the frequency of cardiac, endocrine/diabetic and renal health outcomes.

**Results:**

Of 331 children, 81.0% had obesity at baseline and 22.7% reported use of weight loss diet or medication at baseline or later. Among participants with ≥2 weight measurements ≥2 years apart (*n* = 186), 17.2% had a higher weight classification from the first to the last assessment. Increasing hyperphagia severity was associated with increasing obesity. The prevalence of cardiac, diabetic, endocrine and renal health outcomes increased with obesity class.

**Conclusion:**

Hyperphagia and obesity are highly prevalent in children with BBS. Many children with BBS also experience adverse health outcomes. Timely diagnosis and targeted treatment of hyperphagia and obesity are needed.

## 1 INTRODUCTION

Bardet‐Biedl syndrome (BBS) is a rare genetic disease associated with melanocortin‐4 receptor (MC4R) pathway dysfunction. BBS is characterized by multiorgan pathology, including hyperphagia (i.e., satiety impairment and pathologic, insatiable hunger accompanied by abnormal food‐seeking behaviours) and early‐onset obesity.^1^, ^2^, ^3^, ^4^, ^5^, ^6^, ^7^ Individuals with BBS also commonly present with retinal degeneration, polydactyly, renal anomalies, genital anomalies and learning difficulties.^1^, ^8^ The hypothalamic MC4R pathway is a key regulator of hunger, satiety and energy expenditure and consequently regulates body weight.^9^, ^10^, ^11^, ^12^ Impaired MC4R signalling associated with primary cilia dysfunction is hypothesized to contribute to the hyperphagia and early‐onset, severe obesity observed in BBS.^3^, ^4^, ^13^

The characteristics of BBS, in particular hyperphagia and obesity, often develop early in childhood, are progressive and cause substantial clinical and quality of life burden for individuals and their caregivers.^1^, ^5^, ^8^, ^14^, ^15^, ^16^, ^17^ Individuals with BBS describe hyperphagia as an all‐consuming and extreme hunger, contributing to both physical and emotional burden.^5^, ^17^ One report of obesity in BBS found that 23% of individuals had obesity before 2 years of age, and this proportion increased to 60% among those aged 2 to 5 years.^18^ Furthermore, obesity severity is associated with the development of cardiac, endocrine/diabetic and renal comorbidities within the overall paediatric population, adding to the overall burden.^19^, ^20^

Studies on the natural history of hyperphagia, weight gain and health outcomes in individuals with BBS to date have been limited. The Clinical Registry Investigating BBS (CRIBBS) is an international registry with an overarching goal to understand and quantify unmet medical needs of individuals with BBS. Previous studies exploring data from CRIBBS have reported on the prevalence and characteristics of obesity, renal failure and transplant and developmental milestone achievement in individuals with BBS.^18^, ^21^, ^22^, ^23^ Data from CRIBBS offer an opportunity for a greater understanding of the natural history of weight gain, as well as the relationship between weight, hyperphagia and health outcomes in individuals with BBS across age groups. These data can support timely diagnosis and management to reduce disease burden. To date, CRIBBS has enrolled >300 participants <18 years old, providing a unique opportunity to gain insights into the early stages of this disease. This study had 3 main objectives: (1) to investigate the natural history of weight gain; (2) to elucidate the severity of hyperphagia and its association with obesity; and (3) to assess the frequency of development of cardiac, endocrine/diabetic and renal health outcomes in paediatric participants with BBS enrolled in CRIBBS.

## 2 MATERIALS AND METHODS

### 2.1 CRIBBS overview and enrolment criteria

CRIBBS is an international registry that collects longitudinal data in individuals with BBS (NCT02329210). The registry was established by the Marshfield Clinic in June 2014 with the goal of gathering comprehensive health information on individuals with BBS in a single repository. Potentially eligible individuals are recruited via family support organizations and an online registration portal. Demographic information, health questionnaires, behavioural surveys and health information are collected from participants and provider facilities by a CRIBBS research coordinator at enrolment and at annual follow‐up assessments via telephone or internet services. Health information is obtained from providers and treatment facilities with appropriate authorization. Certain data are collected at enrolment only (e.g., demographic information, family history), while for other data, the CRIBBS research coordinator conducts annual updates of health information and behavioural health surveys.

Individuals eligible for enrolment in CRIBBS are those with ≥1 of the following: (1) manifestation of 4 primary characteristics of BBS^1^; (2) manifestation of 3 primary and 2 secondary characteristics of BBS; (3) genetic confirmation of homozygosity (i.e., homozygous or compound heterozygous) for an established gene variant associated with BBS. Verbal informed consent was obtained from all participants and/or legal guardians enrolled in CRIBBS. The study was conducted according to the Declaration of Helsinki and approved by the Marshfield Clinic Research Foundation Institutional Review Board (IRB‐18‐164).

### 2.2 Measurements and outcomes

#### 2.2.1 Weight and weight loss interventions

The first objective of this analysis was to assess the natural history of weight gain as well as weight loss intervention patterns in children with BBS enrolled in CRIBBS. Self‐reported weight, height and weight loss interventions were evaluated. Weight categories were defined on the basis of the percent of the 95th percentile for body mass index (%BMI95) for age and sex per Centers for Disease Control and Prevention (CDC) growth charts for ages 2 to 20 years. Weight categories included underweight (<5th BMI percentile), normal weight (≥5th to <85th BMI percentile), overweight (≥85th to <95th BMI percentile), class I obesity (%BMI95 ≥ 95% to <120%), class II obesity (%BMI95 ≥ 120% to <140%) or class III obesity (%BMI95 ≥ 140%).^24^, ^25^ For participants <3 years of age at enrolment, the weight category was determined by the first weight measurement after the age of ≥3 years using CDC growth charts.

Weight loss interventions included weight loss diets (low calorie, low carbohydrates, low sugar, low fat, high protein and/or established weight loss diets [such as South Beach Diet or Jenny Craig]), weight loss medications (bupropion, liraglutide, metformin, phendimetrazine, phentermine and semaglutide) and weight loss surgery. Because setmelanotide was an investigational agent during the study period, participants receiving setmelanotide were censored at treatment start. Current weight loss diets/medications were self‐reported at enrolment and at each annual assessment. Weight loss surgery data were collected both historically (procedures before CRIBBS enrolment) and between consecutive assessments. Participants recruited before the age of 3 years who reported current use of weight loss diet/medications before but not after the age of 3 years were counted as users of weight loss diet/medications during the study period.

#### 2.2.2 Hyperphagia

The second objective of this analysis was to evaluate the severity of hyperphagia and the relationship between hyperphagia and obesity. Hyperphagia was assessed using the Hyperphagia Questionnaire, a 13‐item questionnaire that includes subscales for behaviour, drive and severity.^6^ Each item is scored on a 5‐point scale, with higher scores indicating more severe symptoms and possible scores ranging from 11 to 55. The questionnaire was originally validated and developed for use in Prader‐Willi syndrome and has also been used to assess hyperphagia severity in patients with monogenic and syndromic forms of obesity.^26^ In CRIBBS, the Hyperphagia Questionnaire was administered to the parents or caregivers of children with BBS at the fourth, sixth and eighth annual assessments after the initial interview. Given the hyperphagia sample size was small for the sixth and eighth annual assessments, this analysis used data collected at the fourth annual assessment only. Weight categories analysed for this objective were also based on weight measurements at the fourth assessment.

#### 2.2.3 Health outcomes

The third objective of this analysis was to describe acquired (i.e., not congenital) cardiac, endocrine/diabetic and renal health outcomes in participants with BBS categorized by weight category at the first assessment with weight measurement (e.g., baseline). After removing diagnoses that were congenital (e.g., horseshoe‐shaped kidney), diagnoses that may have been congenital (e.g., heart valve problems), or diagnoses with data quality issues (e.g., kidney stones), the following diagnoses were retained for the current CRIBBS outcome analysis: (1) cardiac outcomes included left ventricular hypertrophy, cardiomyopathy, myocardial infarction and heart surgery; (2) renal outcomes included kidney disease, diabetes insipidus, vesicoureteral reflex, short‐term kidney failure, long‐term kidney failure, long‐term dialysis and kidney transplant; (3) endocrine outcomes included Graves' disease, Hashimoto thyroiditis, adrenal and pituitary gland disorders, and other thyroid problems requiring medication; (4) diabetic outcomes were defined as diabetes mellitus and evaluated separately from other endocrine outcomes. At enrolment, both history of and current health outcomes were reported; outcomes reported during annual assessments included new occurrences since the last assessment.

### 2.3 Study populations and analytic approach

#### 2.3.1 Study sample

The study sample included children (age < 18 years at enrolment) with ≥1 weight/height assessment after the age of 3 years who had enrolled in CRIBBS as of June 2021. Descriptive statistics for BMI/weight category at the first weight assessment at the age of ≥3 years and reported weight loss interventions across all assessments in CRIBBS before the age of 18 years are reported for the overall study sample, along with participant demographic characteristics at enrolment. Different study cohorts were included for the analysis of each of the 3 study outcomes as described subsequently and based on the availability of data for each outcome. Additional information on health insurance coverage and education level was evaluated. Expected education level was evaluated on the basis of age at enrolment (e.g., ages 6–8 years, expected first grade level; ages 7–9 years, expected second grade level).

#### 2.3.2 Natural history cohort

The natural history cohort was defined as children aged <18 years with ≥2 weight measurements after the age of ≥3 years that were ≥2 years apart to capture long‐term changes in weight. The natural history of weight gain was assessed as the change in weight category between the first and last weight assessments in children in the natural history cohort.

#### 2.3.3 Hyperphagia cohort

The hyperphagia cohort comprised participants with ages ranging from ≥3 to <18 years with caregiver‐completed Hyperphagia Questionnaire data at the fourth annual assessment after enrolment and had a weight measurement at the assessment or in the year before the assessment. Weight categories were inferred from the most recent weight measurement from 1 year before until the fourth annual assessment, inclusive, so that the gap between the hyperphagia assessment and weight measurement did not exceed 1 year. Pearson's correlation was used to examine the relationship between Hyperphagia Questionnaire score and BMI percentile and %BMI95 at the fourth annual assessment.

#### 2.3.4 Health outcomes cohort

The health outcomes cohort included children among the overall study sample without any indicator of congenital renal or cardiac conditions at enrolment or during the subsequent annual assessments. Descriptive analyses of the proportion of paediatric participants with BBS with any indicators of acquired cardiac, endocrine/diabetic or renal outcomes at enrolment or in any subsequent CRIBBS assessment before the age of 18 years, including diagnoses before CRIBBS enrolment, were categorized by weight category at the first weight assessment after the age of 3 years.

## 3 RESULTS

### 3.1 CRIBBS participant demographics and characteristics

Overall, 331 children with BBS (51.4% male; median age, 9.0 [interquartile range (IQR), 4.0–13.0] years) were enrolled in CRIBBS between 2014 and 2021 and had ≥1 weight/height assessment between the ages of ≥3 and < 18 (Table 1). The median number of follow‐up assessments was 3.4 (range, 1–8). In this international sample, most of the enrolled participants were from the United States (73.4%). For participants in the United States, more had private versus public health insurance (49.0% vs. 34.2%; 15.2% had both), reflecting proportions for the general population (66% public vs. 36% private in 2022).^27^ Overall, 65% of all participants reported initiating education grade levels as expected for age. At the first available weight measurement after the age of ≥3 years (i.e., baseline), 81.0% (*n* = 268) of all children had obesity; 26.0% (*n* = 86), 24.2% (*n* = 80) and 30.8% (*n* = 102) had class I, II and III obesity, respectively. Overall, 22.7% of all paediatric participants (*n* = 75) reported use of a weight loss diet/medication from enrolment to the last available assessment. All patients who used weight loss interventions before the age of 3 years (*n* = 5; first reported median [IQR] CDC BMI percentile, 98.8 [70.0–98.9]) had additional intervention after the age of 3 years. All 5 patients received dietary weight loss intervention before the age of 3 years; none reported use of medications. No participants reported use of weight loss surgery before or during the study period. Metformin was the most frequently reported weight loss medication (7.3% [*n* = 24]). Class III obesity at baseline was overrepresented among children who reported using weight loss interventions during the study period (41.3% [*n* = 31]).

**TABLE 1:** Demographics and baseline characteristics of the overall population.

	Overall population (*N* = 331)
Age at enrolment, mean (median) [IQR], y	8.7 (9.0) [4.0–13.0]
Sex, *n* (%)	
Male	170 (51.4)
Female	161 (48.6)
Race, *n* (%)	
White	243 (73.4)
Black	11 (3.3)
Asian	22 (6.6)
Other^a^	53 (16.0)
Unknown	2 (0.6)
Ethnicity	
Hispanic or Latino	36 (10.9)
Not Hispanic or Latino	295 (89.1)
Region, *n* (%)	
North America	264 (79.8)
United States	243 (73.4)
Canada	19 (5.7)
Unknown	2 (0.7)
Europe	35 (10.6)
Australia	18 (5.4)
Asia	5 (1.5)
Africa	4 (1.2)
Oceania	3 (0.9)
Unknown	2 (0.6)
Education, *n* (%)^b^	
Behind expectation	7 (2.1)
As expected	215 (65.0)
Ahead of expectation	6 (1.8)
Missing	18 (5.4)
Not applicable	85 (25.7)
Type of healthcare insurance, *n* (% of children in the United States)	
Public	83 (34.2)
Private	119 (49.0)
Both public and private	37 (15.2)
Unknown	4 (1.6)
Weight category^c^ at first weight assessment from age ≥3 to <18 years, *n* (%)	
Underweight	1 (0.3)
Normal weight	32 (9.7)
Overweight	30 (9.1)
Obesity	268 (81.0)
Class I	86 (26.0)
Class II	80 (24.2)
Class III	102 (30.8)
CDC BMI Z score at first weight assessment, mean (median) [IQR]^d^	2.3 (2.4) [1.9–2.8]
CDC BMI percentile at first weight assessment, mean (median) [IQR]^d^	94.5 (99.2) [97.1–99.8]
Weight loss interventions reported from enrolment to the last assessment, *n* (%)^e^	
Diet only^f^	49 (14.8)
Medication only^g^	22 (6.6)
Diet + medication	4 (1.2)
Surgery ± diet or medication	0
Weight loss medication currently used across all annual assessments, *n* (%)^h^	
Metformin	24 (7.3)
Bupropion	2 (0.6)
Liraglutide	0
Phendimetrazine	0
Phentermine	0
Semaglutide	0
Weight category^c^ at first weight assessment in participants reporting use of weight loss diet/medication, *n* (%)^i^	
Underweight	0
Normal weight	7 (9.3)
Overweight	7 (9.3)
Obesity	61 (81.3)
Class I	15 (20.0)
Class II	15 (20.0)
Class III	31 (41.3)

Abbreviations: BMI, body mass index; CDC, Centers for Disease Control and Prevention; IQR, interquartile range; WHO, World Health Organization.

^a^ Middle Eastern, American Indian or Alaska Native, and Pacific Islander.

^b^ Based on responses to the question, ‘If the participant is less than 18 years old, what is the highest grade the participant has started?’

^c^ Defined on the basis of %BMI95 for age and sex per CDC growth charts: underweight (<5th BMI percentile), normal weight (≥5th to <85th BMI percentile), overweight (≥85th to <95th BMI percentile), class I obesity (%BMI95 ≥ 95% to <120%), class II obesity (%BMI95 ≥ 120% to <140%), class III obesity (%BMI95 ≥ 140%).

^d^ BMI Z scores and BMI percentiles calculated according to CDC growth charts.

^e^ For participants recruited before the age of 3 years, those currently using weight loss diet/medications before but not after the age of 3 years were counted as users of weight loss diet/medications during the study period.

^f^ Multiple diets possible.

^g^ Multiple medications possible.

^h^ Weight loss medications take into account all historic and new uses from enrolment to the last annual assessment with or without other weight loss interventions, and participants who received investigational medication in the context of a clinical trial were censored at the start of treatment.

^i^ Percentages reported among 75 children with weight loss intervention use. %BMI95, percent of the 95th percentile for BMI.

### 3.2 Natural history of obesity in BBS

**FIGURE 1:**
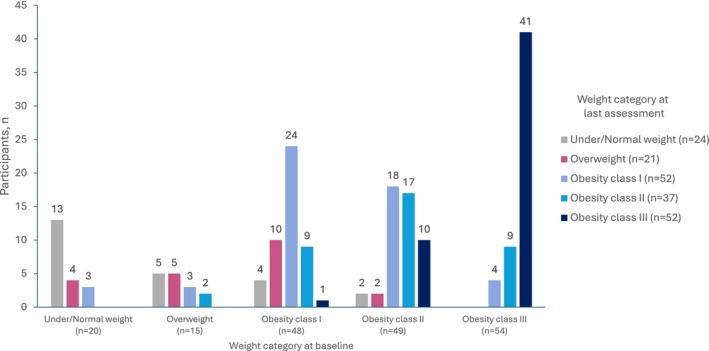
Natural history of change in weight category from baseline to last assessment. [Correction added on 14 June 2025, after first online publication: The x‐axis label in figure 1 was incorrect and has been corrected in this version.]

A total of 186 children were included in the natural history cohort. Of these, 81.2% (*n* = 151) had obesity at baseline. Overall, 17.2% of children (*n*/*N* = 32/186) moved to a higher weight category by the last annual assessment available ≥2 years later; 4.3% (*n*/*N* = 8/186) moved from below to above the obesity threshold, and 10.8% (*n*/*N* = 20/186) moved from one obesity class to a higher one (e.g., class I to class II; Figure 1). Among participants with obesity, 10 of 24 with obesity class I developed obesity class II or III, and 10 of 17 with obesity class II developed obesity class III between the first and last assessment. In addition, 29% (*n*/*N* = 54/186) went from being in a higher weight category to being in a lower one, including 18 participants who went from having obesity at baseline to being overweight or normal/underweight at the last assessment. No clear trends in baseline demographics were observed in these 18 participants; none reported use of weight loss medication or surgery, and 5 reported use of dietary intervention. Overall, 53.8% (*n*/*N* = 100/186) did not have a change in weight categories between assessments, including 41 participants who already had obesity class III at the initial assessment.

### 3.3 Hyperphagia burden and association with obesity

**FIGURE 2:**
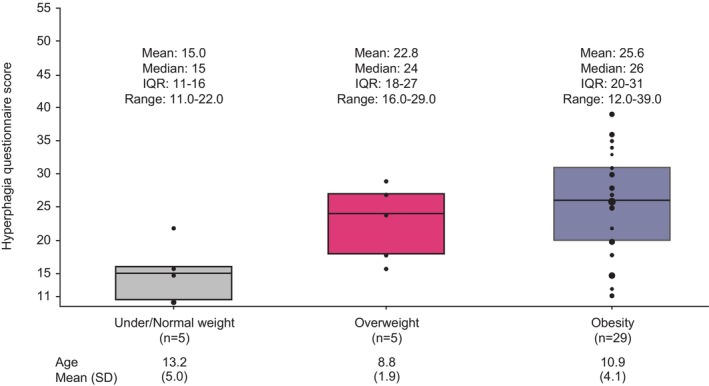
Hyperphagia scores by weight category. Horizontal lines inside boxes are the median, boxes are the IQR and black circles are the individual data, with increasing circle size representing increasing number of participants with the same value. Hyperphagia and weight categories reported here are from the fourth annual assessment. IQR, interquartile range; SD, standard deviation.

**FIGURE 3:**
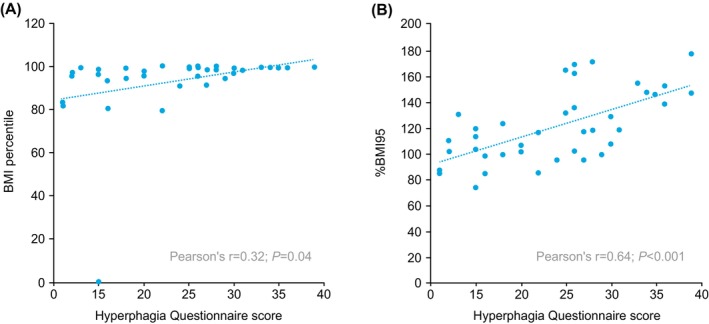
Relationship between hyperphagia and obesity quantified by (A) BMI percentile or (B) %BMI95. Each data point indicates 1 participant in the sample of 39 children in the hyperphagia cohort. Lines are trend lines illustrating the average increase in BMI percentile or %BMI95 per point increase in hyperphagia score. The trend lines were estimated from ordinary least squares regression models, with BMI percentile and %BMI95 as dependent variables and hyperphagia score as the independent variable. Hyperphagia and weight categories reported here are from the fourth annual assessment. BMI, body mass index; %BMI95, percent of the 95th percentile for BMI.

The Hyperphagia Questionnaire was completed by caregivers of 39 participants with BBS at the fourth annual assessment (61.5% male; mean age, 10.9 years [median, 11.0 years]). The mean BMI percentile for participants in this population was 93.6 (median, 98.5; IQR, 94.5–99.6); 5 participants had under/normal weight, 5 had overweight, and 29 had obesity at the fourth annual assessment. Across all children, a mean Hyperphagia Questionnaire score of 23.9 (median, 25.0; IQR, 16.0–30.0) was reported by caregivers. The Hyperphagia Questionnaire score generally increased by weight category (Figure 2) and was significantly positively correlated with BMI percentile (*r* = 0.32; *p* = 0.04) (Figure 3A) and with %BMI95 (*r* = 0.64; *p* < 0.001) (Figure 3B).

### 3.4 Prevalence of Health Outcomes

A total of 318 children were included in the health outcomes cohort (50.6% male; mean age, 8.7 years [median 9.0 years]). The mean BMI percentile at the first assessment for this population was 94.7 (median, 99.3); 31 children (9.7%) had underweight/normal weight, 29 (9.1%) had overweight, 81 (25.5%) had class I obesity, 77 (24.2%) had class II obesity, and 100 (31.4%) had class III obesity.

**FIGURE 4:**
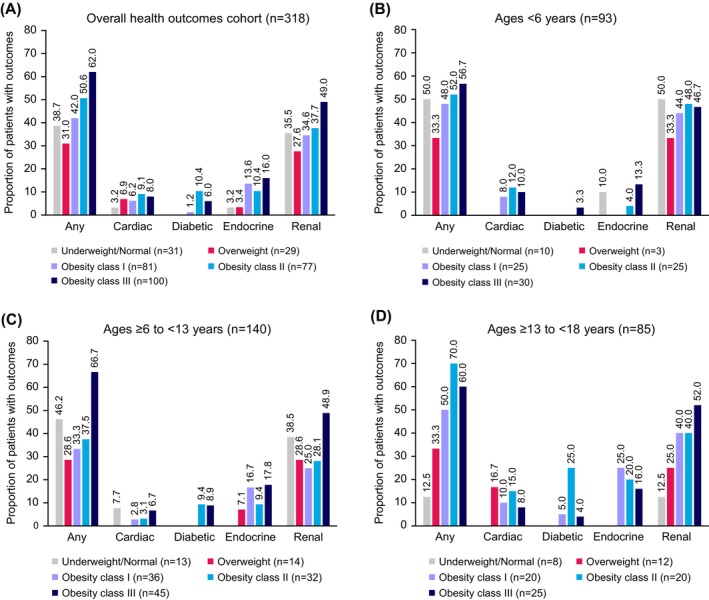
Prevalence of health outcomes by weight category in (A) the overall health outcomes cohort, (B) participants aged <6 years, (C) participants aged ≥6 to <13 years and (D) participants aged ≥13 to <18 years.

Of the 318 children included in the health outcomes cohort, 49.1% (*n* = 156) had indicators for a health outcome of interest, either at enrolment (i.e., historic diagnosis) or during an annual assessment (i.e., new diagnosis). The prevalence of any cardiac, endocrine, diabetic or renal outcome increased with obesity class at the first weight measurement after age ≥3 years after enrolment (Figure 4). Overall, renal outcomes were reported for 125 children (39.3%), endocrine outcomes for 37 (11.6%), cardiac outcomes for 23 (7.2%) and diabetic outcomes for 15 (4.7%). Reported incidences of renal outcomes were 34.0% for kidney disease, 13.8% for vesicoureteral reflux, 6.9% for long‐term kidney failure, 4.1% for diabetes insipidus, 3.1% for kidney transplant and 2.8% each for short‐term kidney failure and long‐term dialysis. For endocrine outcomes, the reported diagnoses were thyroid problems requiring medication (7.2%), pituitary gland problems (3.1%), Hashimoto's thyroiditis (2.2%) and adrenal gland problems (1.3%). Abnormal echocardiogram was the most frequently reported cardiac outcome (6.6%), followed by heart valve issues (4.4%), cardiomyopathy (2.2%), left ventricular hypertrophy (0.9%) and heart surgery (0.6%).

Cardiac, endocrine, diabetic and renal diagnoses were generally first observed in early childhood. Among participants 0 to <6 years of age (*n* = 93), outcomes were reported in 50.0%, 33.3%, 48.0%, 52.0% and 56.7% of those with underweight/normal weight, overweight, class I obesity, class II obesity and class III obesity, respectively. Among participants <2 years old at enrolment, 73% with obesity had experienced ≥1 health outcome before or after enrolment (*n*/*N* = 19/26; Table 2).

**TABLE 2:** Health outcomes in participants <2 years old at enrolment (*n* = 32).

	Underweight/Normal weight (*n* = 5)	Overweight (*n* = 1)	Obesity class I (*n* = 9)	Obesity class II (*n* = 8)	Obesity class III (n = 9)
Participants with outcome, indicators (at enrolment or later), n (%)	3 (60.0)	0	6 (66.7)	5 (62.5)	8 (88.9)
Participants with ≥1 outcome diagnosis before enrolment	1 (33.3)	0	5 (83.3)	3 (60.0)	2 (25.0)
Participants whose outcomes were diagnosed after enrolment	2 (66.6)	0	1 (16.7)	2 (40.0)	6 (75.0)

## 4 DISCUSSION

Hyperphagia is a severe manifestation and hallmark of BBS that affected individuals and caregivers note as being highly distressing and conferring negative impacts on quality of life.^5^, ^15^, ^16^, ^18^ The increased energy intake driven by hyperphagia can contribute to obesity.^7^, ^28^ In the current study, a significant positive correlation between the severity of hyperphagia and BMI percentile and %BMI95 was observed. Furthermore, obesity in paediatric individuals with BBS is highly prevalent, occurring in >80% of the participants evaluated in CRIBBS and persisting over time, despite some individuals using obesity management strategies. These data are aligned with previous reports of obesity prevalence in individuals with BBS ranging from 72% to 92%.^29^, ^30^

Hyperphagia in BBS negatively impacts both affected individuals and their caregivers.^16^, ^17^, ^18^ While the Hyperphagia Questionnaire used in this study was developed in individuals with Prader‐Willi syndrome, it has been used in other populations to assess hyperphagia severity.^6^, ^31^ One study including 13 individuals with BBS found significantly greater Hyperphagia Questionnaire total and subscale scores in patients with BBS compared with controls (*n* = 23).^5^ Among evaluable participants in CRIBBS, the mean caregiver‐reported hyperphagia score was 23.9, supporting previous reports of severe hyperphagia in BBS. Measuring hyperphagia in clinical settings can be challenging because of a lack of validated tools as well as the subjectivity of hunger ratings.^14^, ^15^, ^16^ Despite these limitations, the current analysis demonstrates hyperphagia is present and severe among participants with BBS enrolled in CRIBBS.

Most participants (71%) in the current study remained at or moved to a higher weight category over time, highlighting the disease trajectory and need for early intervention to mitigate weight gain. Additionally, 29% of participants went from being in a higher weight category to being in a lower one between the first and last follow‐up. This observation may not indicate weight loss but instead weight gain at a rate resulting in a lower BMI percentile for age and sex as the individual ages, grows and their height increases. In a previous analysis of paediatric and adolescent participants in CRIBBS, similar trajectories of lower BMI Z score with age among participants 6–11 years compared with those 2–5 years were observed, with increasing overall prevalence of obesity.^18^ This finding may be partly associated with the motivation and engagement of registry participants and their families to understand the disease and how to approach management at home. Indeed, in other rare forms of obesity, diagnosis alone can empower caregivers to cope with stigma and optimize supportive treatment.^32^

Early‐onset obesity is critical to address, because obesity in childhood is associated with increased risk of cardiovascular and metabolic comorbidities in adulthood, along with increased stigma and burden.^28^, ^33^ There are limited data examining the impacts of early‐onset obesity on long‐term health risks in individuals with BBS, although work in general obesity may provide insights into the risks. A recent study of 140 paediatric individuals with obesity found a high prevalence of cardiovascular risk factors, including elevated blood pressure, low high‐density lipoprotein cholesterol and high triglycerides, with 76% of individuals showing ≥2 metabolic complications.^20^ These individuals also demonstrated an increased severity of metabolic syndrome with increased age, highlighting the need for early intervention to mitigate future complications. In the present study, paediatric participants with BBS and obesity generally demonstrated an increased prevalence of cardiac, endocrine/diabetic and renal outcomes than children without obesity, adding to their clinical burden. It is important to note that some of these health outcomes may be associated with BBS disease pathology itself rather than obesity alone. For instance, renal anomalies, congenital heart disease and diabetes mellitus are hallmarks of BBS and are used for clinical diagnosis.^30^, ^34^ The interplay between disease progression, which itself includes early‐onset obesity, and severity of obesity impacting these health outcomes should be considered because some outcomes might not be directly related to but could be exacerbated by obesity (e.g., congenital heart disease and renal dysfunction). Early identification and intervention in individuals with genetic forms of obesity, such as BBS, is paramount because the presence of pathogenic variants from birth can confer lifelong impacts on weight.^4^, ^35^ Novel treatments for managing weight and hunger in BBS have emerged in recent years, providing meaningful therapy for a disease that is typically refractory to traditional management strategies.^30^, ^36^ The MC4R agonist setmelanotide has demonstrated clinically meaningful reductions in body weight and hunger in patients with BBS as young as 2 years old and is approved for use in this population in the United States and European Union.^37^, ^38^ Limited evidence is available for the effect of glucagon‐like peptide‐1 receptor agonist therapy in individuals with BBS for weight and hunger management, although 1 case study reported successful weight reduction with dietary management in combination with liraglutide and semaglutide in an adult with BBS.^39^, ^40^ The availability of effective treatments for obesity in BBS underscores the importance of early diagnosis in order to circumvent further weight gain and risk of potential comorbidities.

Considering the multisystem dysfunction and predisposition to hyperphagia and early‐onset, severe obesity, it is important to distinguish BBS and other genetic causes of obesity from general obesity. Individuals with BBS are often refractory to traditional weight loss strategies such as lifestyle modifications and bariatric surgery, potentially owing to hyperphagia and the underlying mechanism of disease, underscoring the need for early identification to ensure treatment with targeted therapy can be explored.^35^, ^36^, ^41^, ^42^ The diagnosis of BBS is often made clinically on the basis of presenting characteristics, although oftentimes, a BBS diagnosis may not occur until problems with vision begin to present later in childhood. Genetic testing can confirm the underlying pathology and inform treatment strategies in most cases.^1^, ^19^ A recent consensus statement recommended the use of genetic screening in the diagnosis of BBS in settings where testing is available.^34^ Differentially diagnosing BBS from general obesity is important not only to ensure adequate management of multisystem characteristics such as endocrine, visual and renal dysfunction, but also to provide tailored hyperphagia and obesity management strategies.^19^, ^36^

A key strength of this analysis is that CRIBBS is the largest global registry currently available; the current analysis comprised 331 participants globally with a range of backgrounds. Limitations of the study include the self‐ and caregiver‐reported nature of weight‐related measures and health outcomes. Anthropometric measurements were primarily obtained at healthcare encounters, with some self‐reported values, and do not reflect the precision of single‐centre prospective studies. Because weight loss interventions were self‐reported, it is possible that routine efforts to manage hunger and overeating were not considered by the participant/caregiver as a weight loss intervention; thus, it is possible the reported weight loss interventions in the current study are undercounted and biased toward high‐intensity interventions. Hyperphagia Questionnaire data are caregiver reported rather than participant reported, and therefore potentially subject to bias. Weight category was classified using CDC growth chart data, and although our study population was primarily made up of individuals from the United States, the growth standards may not be applicable to the global population. The current analysis also did not evaluate any genotype–phenotype correlations; thus, conclusions on the contribution of individual *BBS* variants to outcomes cannot be made. As noted previously, some of the evaluated health outcomes could be driven by BBS, obesity or both; the current analysis was not designed to delineate between what outcomes were specifically BBS related versus obesity related. Furthermore, some outcomes can be either congenital or noncongenital (e.g., vesicoureteral reflux), which was difficult to differentiate from the self‐reported data. Finally, the current analysis reports cross‐sectional data for hyperphagia and health outcomes rather than longitudinal data. CRIBBS is continuing to collect data, and future studies evaluating longitudinal changes should be assessed when a sufficient sample size is achieved.

## 5 CONCLUSION

Obesity is highly prevalent early in life in children with BBS, and most children experience continued weight gain despite the use of obesity management strategies. Hyperphagia in children with BBS is generally severe and increases with obesity class; management strategies that target the underlying pathology of hyperphagia may prove effective in this population. This analysis further shows that a large proportion of individuals with BBS and severe obesity experience cardiac, endocrine and renal health outcomes early in life. Hyperphagia and obesity are early manifestations in BBS, which may support earlier diagnosis and management of the overall disease. Additional research on the extent to which obesity is involved in the development or severity of these outcomes, which are often associated with BBS, is needed. Given the comprehensive clinical burden that paediatric individuals with BBS experience as it relates to hyperphagia, obesity, and other health outcomes, there is substantial unmet need for early identification and appropriate management. Timely diagnosis and early implementation of hyperphagia and weight management strategies in paediatric individuals with BBS may subsequently reduce the burden associated with cardiac, endocrine/diabetic and renal health outcomes.

## AUTHOR CONTRIBUTIONS

RMH and JP designed the CRIBBS program. CH conceptualized the analysis in the present study. JP, UO, JGR and RMH were involved in the acquisition of data in CRIBBS. AF, RI‐I and FV analysed the data. All authors were involved in drafting, critically reviewing and providing final approval of the manuscript.

## FUNDING INFORMATION

Funding for this study was provided by Rhythm Pharmaceuticals, Inc. Writing and editorial assistance was provided under the direction of the authors by Rhyomi Sellnow, PhD, CMPP, and David Boffa, ELS, of MedThink SciCom, and funded by Rhythm Pharmaceuticals, Inc.

## CONFLICT OF INTEREST STATEMENT

JP receives research support from Rhythm Pharmaceuticals, Inc., is a coinvestigator for Rhythm clinical trials and is a coinvestigator of a study examining unmet medical needs related to obesity in people with BBS. UO has received payment as a speaker for educational events/lectures from Rhythm Pharmaceuticals, Inc. JP, UO, JGR and RMW's institution have received funding for research from Rhythm Pharmaceuticals, Inc. AF, RI‐I and FV are employees of STATLOG Inc., a consultancy company that received funding from Rhythm Pharmaceuticals, Inc. to conduct this study. CH is employed by and receives stock options from Rhythm Pharmaceuticals, Inc.
